# Towards Automated Model Selection for Wind Speed and Solar Irradiance Forecasting

**DOI:** 10.3390/s24155035

**Published:** 2024-08-03

**Authors:** Konstantinos Blazakis, Nikolaos Schetakis, Paolo Bonfini, Konstantinos Stavrakakis, Emmanuel Karapidakis, Yiannis Katsigiannis

**Affiliations:** 1 School of Electrical and Computer Engineering, Technical University of Crete, 73100 Chania, Greece; 2 QUBITECH, Quantum Technologies, 15231 Athens, Greece; 3 Institute of Computational Mechanics and Optimization, School of Production Engineering and Management, Technical University of Crete, 73100 Chania, Greece; nischetakis@tuc.gr; 4 Quantum Innovation Pc, 73100 Chania, Greece; pbo@alma-sistemi.com; 5 Alma-Sistemi Srl, IT-00012 Guidonia, Italy; 6 Department of Quantum and Computer Engineering, Delft University of Technology, 2628 Delft, The Netherlands; k.stavrakakis@tudelft.nl; 7 Department of Electrical and Computer Engineering, Hellenic Mediterranean University, 71410 Heraklion, Greece; karapidakis@hmu.gr (E.K.); katsigiannis@hmu.gr (Y.K.)

**Keywords:** artificial intelligence, data mining, deep learning, machine learning, wind speed forecasting, solar irradiance forecasting, increased RES penetration, smart grids

## Abstract

Given the recent increase in demand for electricity, it is necessary for renewable energy sources (RESs) to be widely integrated into power networks, with the two most commonly adopted alternatives being solar and wind power. Nonetheless, there is a significant amount of variation in wind speed and solar irradiance, on both a seasonal and a daily basis, an issue that, in turn, causes a large degree of variation in the amount of solar and wind energy produced. Therefore, RES technology integration into electricity networks is challenging. Accurate forecasting of solar irradiance and wind speed is crucial for the efficient operation of renewable energy power plants, guaranteeing the electricity supply at the most competitive price and preserving the dependability and security of electrical networks. In this research, a variety of different models were evaluated to predict medium-term (24 h ahead) wind speed and solar irradiance based on real-time measurement data relevant to the island of Crete, Greece. Illustrating several preprocessing steps and exploring a collection of “classical” and deep learning algorithms, this analysis highlights their conceptual design and rationale as time series predictors. Concluding the analysis, it discusses the importance of the “features” (intended as “time steps”), showing how it is possible to pinpoint the specific time of the day that most influences the forecast. Aside from producing the most accurate model for the case under examination, the necessity of performing extensive model searches in similar studies is highlighted by the current work.

## 1. Introduction

The use of fossil fuels constitutes a substantial portion of both national and worldwide energy requirements. The utilization of resources such as oil, coal, and natural gas has been scientifically established to emit substantial quantities of greenhouse gases into the atmosphere, resulting in severe adverse effects on the climate. Embracing carbon-free, renewable energy sources such as wind and solar power, which have begun to be harnessed in the last few decades in order to meet the increasing global energy demands, offers the opportunity to produce energy that is more environmentally friendly. The opening up of the electric energy market and the growing demand for sustainable energy have influenced both governmental and financial investment strategies in promoting the increased adoption of RESs in order to meet the demands for power in a more environmentally responsible manner [1,2].

The quantity of energy produced from wind and solar sources is significantly impacted by various local weather variables such as temperature, wind speed, air pressure, humidity, sunlight, and their fluctuations. Consequently, effectively managing and predicting wind and solar power generation presents a challenge due to the continuous variability in weather conditions. This inherent variability makes the integration of wind and solar energy into power grids a complex task, especially within isolated systems [3,4].

To enhance the ability to predict the amount of renewable energy that can be generated in various operational scenarios for the electric grid, it is imperative to enhance the accuracy of one-day-ahead forecasts for wind speed and solar irradiance. Developing computational models that can precisely forecast solar irradiance and wind speed on short-/medium-term time scales is essential, given the intrinsic correlation between solar irradiance and electricity generation from photovoltaic systems, as well as the connection between wind speed and wind turbine power generation [5,6,7,8]. For the purpose of preserving grid stability, enhancing economic efficiency, integrating renewable energy, organizing operations, minimizing environmental effects, and assisting with market and policy development, short-/medium-term wind and solar power forecasting is crucial.

The main goal of this study was to create highly accurate medium-term forecasting models for wind speed and solar irradiance for the next 24 h. This research investigated the efficacy of various model configurations in order to accomplish this goal. The objective was to demonstrate the enhanced predictive power of deep learning methods and determine which was the most effective. The study assessed various approaches with a benchmarked dataset of actual measurements. This research aimed to contribute valuable insights into enhancing the precision of medium-term renewable energy forecasts, facilitating better planning and management of energy resources.

The most important contributions of the present work can be summarized as follows:

- A variety of different model configurations were constructed and employed to generate wind speed and solar irradiance forecasts. A decisive evaluation of these forecasting methods was conducted with the calculation of error indices.
- An extensive feature importance analysis was performed, in order to reveal which predictor and time step (feature) was more important for wind speed and solar irradiance forecasting time steps. The main goal of this study was to create highly accurate medium-term forecasting models, particularly with a focus on wind speed and solar irradiance for the next 24 h. This research investigated the efficacy of various model configurations in order to accomplish this goal. The objective was to demonstrate the enhanced predictive power of deep learning methods and determine which was the most effective. The study assessed various approaches with a benchmarked dataset of actual measurements.
- Linear regression (LR), despite its simplicity, was found to be sufficient for the purposes of wind speed forecasting, yielding performances close to those of the more sophisticated LightGBM and WaveNet algorithms.

The remainder of this article is organized as follows: Section 2 provides a summary of the relevant research; Section 3 presents the dataset utilized for this survey; Section 4 presents the model selection framework and assessment; Section 5 shows the forecasting results of the proposed methods; and finally, Section 6 provides the conclusions and future perspectives.

## 2. Related Research Work

Over the past few years, numerous algorithms have emerged for the prediction of solar irradiance and wind speed [3,6,8]. These forecasting methods can be broadly classified into three primary categories:

- Data-Driven Models:
  This category encompasses statistical models and machine learning models, and it is the most commonly employed set of tools for forecasting time series data. These methods offer various approaches to predicting solar irradiance and wind speed, each having its own strengths and suitability for different forecasting scenarios.
  In the realm of data-driven models, statistical methods encompass a range of techniques, including the auto-regressive moving average (ARMA) [9,10,11], Lasso [12], and Markov models [13,14,15].
  On the other hand, the most commonly used machine learning methods for forecasting solar irradiance and wind speed include support vector machines (SVMs) [16,17], feed forward neural networks (FFNNs) [18], recurrent neural networks (RNNs) [19,20,21], convolutional neural networks (CNNs) [22], long short-term memory networks (LSTMs) [23,24,25,26], bidirectional long short-term memory neural networks (BiLSTMs) [27], deep belief networks (DBNs) [28], artificial neural networks in general (ANNs) [29,30,31], and transformers [32,33].
- Physics-based Models:
  These models rely on meteorological and topographical data to make predictions, taking into account the fundamental principles governing the behavior of these variables. These physics-based methods leverage the understanding of meteorological and atmospheric phenomena to improve the accuracy of renewable energy forecasting.
  In the category of physics-based methods for forecasting solar irradiance and wind speed, several approaches are employed, including numerical weather prediction (NWP) forecasting models [34,35], total sky imagery (TSI) [36], cloud-moving-based satellite imagery models [37], and weather research and forecasting (WRF) models [38].
- Hybrid Algorithms:
  Hybrid algorithms combine elements from both data-driven and physics-based models. They have demonstrated considerable success in various research domains, offering a combination of data-driven flexibility and physical accuracy. These hybrid methods leverage a combination of techniques to enhance the accuracy of solar irradiance and wind speed forecasts, making them valuable tools in renewable energy planning and management.
  In the literature, various hybrid methods have been developed for forecasting solar irradiance and wind speed. Here are some examples: variational mode decomposition with Gram–Schmidt orthogonal and extreme learning machines enhanced by a gravitational search algorithm [39]; nonlinear neural network architectural models combined with a modified firefly algorithm and particle swarm optimization (PSO) [40]; hybrid model decomposition (HMD) method and online sequential outlier robust extreme learning machine (OSORELM) [41]; empirical mode decomposition and Elman neural networks (EMD-ENN) [42]; wavelet transform (WT-ARIMA) [43]; empirical wavelet transform (EWT) and least-square support vector machines (LSSVMs) improved by coupled simulated annealing [44]; complementary ensemble empirical mode decomposition (CEEMD) preprocessing with extreme learning machines (ELMs) and Elman neural networks (ENNs) [45]; sample entropy and VMD forecasting methods based on ENNs and a multi-objective “Satin Bowerbird” optimization algorithm [46]; bidirectional long short-term memory neural networks with an effective hierarchical evolutionary decomposition technique and an improved generalized normal distribution optimization algorithm [47]; combined model system with improved hybrid time series decomposition strategy (HTD), multi-objective binary backtracking search algorithm (MOBBSA), and advanced sequence-to-sequence (Seq2Seq) predictor for wind speed forecasting [48]; and recurrent neural network prediction algorithms combined with error decomposition correction methods [49].

The abundance of models developed throughout the years highlights the difficult challenges posed by the task of energy forecasting. The evident absence of a single prevailing technique constitutes the underlining motivation for this study: to undertake a comprehensive approach by evaluating a collection of models in order to select the most suitable for a specific dataset in an auto-machine learning (ML) fashion.

## 3. Dataset Presentation

The dataset utilized for this study originated from measurements conducted at the Laboratory of Energy and Photovoltaic Systems (LEPS) of Hellenic Mediterranean University (HMU) in Heraklion, Crete, Greece. Table 1 provides parameters recorded at 5 min intervals for each day throughout a one-year period. These measurements were taken at a height of 10 m above the ground using a Campbell Scientific wired weather station. It is worth noting that all parameters of interest were recorded directly, except for diffuse irradiance on the horizontal plane, which was estimated using the anisotropic model as detailed in [50]. Additionally, extraterrestrial irradiance on a horizontal plane was calculated based on standard solar geometry equations, as presented in [51].

Table 2 contains statistical information for several key variables, including global irradiance on the horizontal plane, wind speed, air temperature, diffuse irradiance on the horizontal plane, and extraterrestrial irradiance on the horizontal plane. These statistics included the maximum and minimum mean values, as well as standard deviations (Std), providing valuable insights into the variability and characteristics of these parameters over the study period. 

Wind speed and solar irradiance forecasting can be categorized into four distinct time intervals according to the bibliography [6,7] as shown in Table 3 and Table 4.

In the context of solar irradiance forecasting, it is customary to consider a normalized discrete index for each hour of the day $NDD\left(h,d\right)$; this was calculated using Equation (1). This calculation relied on data derived from two sources: extraterrestrial solar irradiance and solar irradiance measured in the horizontal plane from the photovoltaic laboratory of Hellenic Mediterranean University [34]. Furthermore, to improve the accuracy of the forecasting models, nighttime values (those associated with zero solar irradiance) were excluded from the initial dataset of measurements. This exclusion was reasonable because nighttime hours did not contribute to solar irradiance forecasting and could be considered non-informative data for this purpose.

In the context of solar irradiance forecasting, the research utilized the following parameters as inputs from the initial measurements’ dataset:

- GHI (global horizontal irradiance): This parameter represents the total solar irradiance received on a horizontal surface, including both direct sunlight and diffuse sky radiation.
- DHI (diffuse horizontal irradiance): DHI refers to the solar irradiance received on a horizontal surface solely from the diffuse sky radiation component, which is the portion of solar radiation that has been scattered by molecules and particles in the atmosphere.
- NDD(h,d) (normalized discrete index for each hour of the day): This characterizes the cloud cover or cloudiness for a specific hour of the day on a given day of the year.
- Hour of the day: The time of day, often represented as an hour value, is used as an input to account for diurnal variations in solar irradiance.

These parameters collectively provide valuable information about the incoming solar radiation, the presence of clouds, and the time of day, which are essential factors for accurate solar irradiance forecasting. The inclusion of NDD(h,d) helps account for the influence of cloud cover on solar irradiance, making the forecasting model more robust and accurate.

The parameter $NDD\left(h,d\right)$ was determined through the following equation:

(1) $$NDD\left(h,d\right)=G_{on,h,d}-G_{sn,h,d}$$

where “d” represents the day of the year, ranging from 1 to 365; “h” represents a specific hour of the day for which the cloud index $NDD\left(h,d\right)$ is being calculated; $G_{on,h,d}$ stands for the normalized extraterrestrial irradiance; and $G_{sn,h,d}$ stands for the normalized surface irradiance.

The normalized extraterrestrial irradiance ($G_{on}$) was derived using well-established equations purely based on geometrical considerations. These equations took into account various parameters, including the solar constant (indicating the flux received on a perpendicular unit area; 1367 W/m^2^), the day of the year, the latitude and longitude of the location, the solar hour angle, and the declination angle of the Sun [51]. To normalize both $G_{on}$ and $G_{sn}$, the maximum values for these parameters over the examined year were used as reference points. 

In the context of wind speed forecasting, the research exclusively utilized the wind speed parameter as an input from the initial dataset, essentially framing this as an “auto-regressive” problem. This meant that the forecasting model relied solely on historical data of wind speed to make predictions about future wind speed values. This single input, along with the appropriate modeling techniques, was employed to forecast wind speed, taking into account patterns and variations in wind behavior over time.

**Figure 1:**
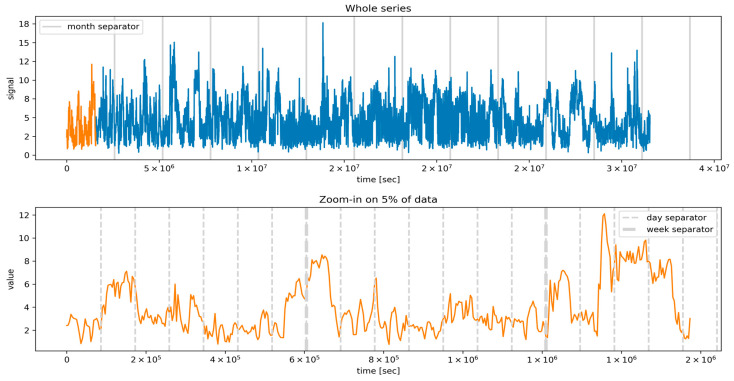
Wind speed time series (m/s). The top panel represents the whole series, while the bottom one shows a zoom-in on a detail (corresponding to the orange subset in the top panel). The x-axis displays the time expressed in seconds from the first entry in the dataset.

**Figure 2:**
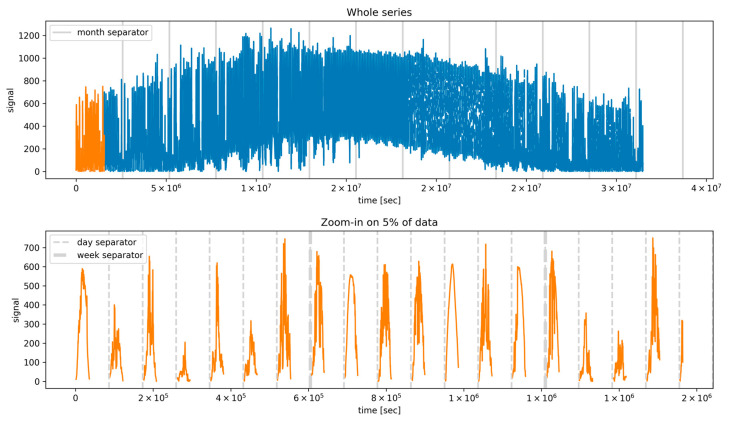
Solar irradiance time series (GHI, W/m^2^). The top panel represents the whole series, while the bottom one shows a zoom-in on a detail (corresponding to the orange subset in the top panel). The x-axis displays the time expressed in seconds from the first entry in the dataset. The gaps represent the nighttime hours, during which the solar irradiance is absent.

Based on the original dataset values resolution of 5 min, an investigation on whether different temporal resolutions would prove more informative was conducted. In order to achieve this, block-averaged values with 10 min, 30 min, and 1 h resolutions were constructed. From a preliminary analysis, the feature corresponding to the 5 min resolution and the 1 h resolution provided the best estimator for solar irradiance and wind speed forecasting, respectively, and it was selected for the analysis presented in the remainder. The choice of different resolutions for the solar and wind cases should not be surprising, since from Figure 1 and Figure 2 (presenting the whole datasets for the wind and solar series), it is clear that the variation of wind speed over time was significantly larger than the solar irradiance. 

In the remainder, the independent variables are referred to as “predictors”, while the term “target” refers to the dependent variable to be forecasted.

## 4. Protocol for Model Selection and Assessment

The best-fit model for either of the wind/solar time series was selected using a cross-validation (CV) technique, and its performance was evaluated over a hold-out test set, which was retained from the last 10% of the data (in temporal order). In the context of time series analysis, a plethora of CV approaches were proposed as there is no universal consensus on which is the ideal methodology that guarantees to account for the temporal correlations inherent in such data [52]. In fact, the challenge for time series CV protocols is that of splicing the data, while making sure of avoiding temporal correlations between the train and test sets, as well as avoiding information leakage (i.e., “peeking into the future”).

**Figure 3:**
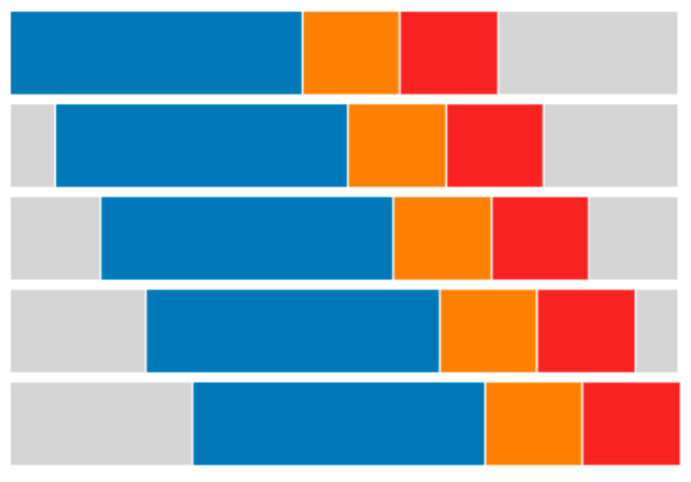
A five-fold rolling origin CV protocol (ROCV), similar to the one we adopted in the current work.

In this study, a conservative protocol was used, known as rolling origin CV (ROCV). In this framework, at each folding, a contiguous fraction of the whole dataset was used, while the remaining fraction was disregarded. The technique is named after the fact that the origin of such a sub-dataset shifts towards later time steps at each folding, as depicted in Figure 3. During a given folding, the sub-dataset was itself split into a training, validation, and test set. Notice that the validation set was exploited in the current work only to evaluate the early stopping of neural network (NN) regressors, yet it was “carved out” (and neglected) for every regressor, so to provide the exact same data pool to each of them. In Figure 3, each row represents a folding, with the whole rectangle representing the full dataset. The blue, orange, and red segments show the fractions of the training, validation, and test sets, respectively. The gray segment represents the fraction of data disregarded for that folding.

**Figure 4:**
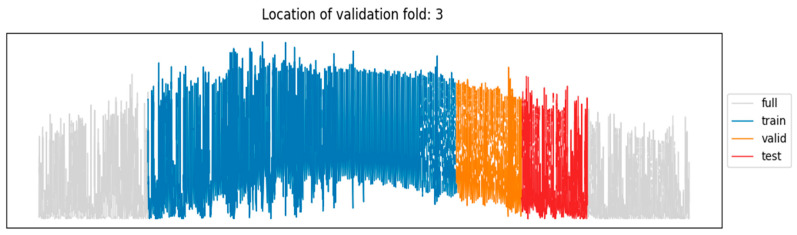
Location of training/validation/test sub-sets (blue/orange/red), along with the whole solar dataset (gray).

**Figure 5:**
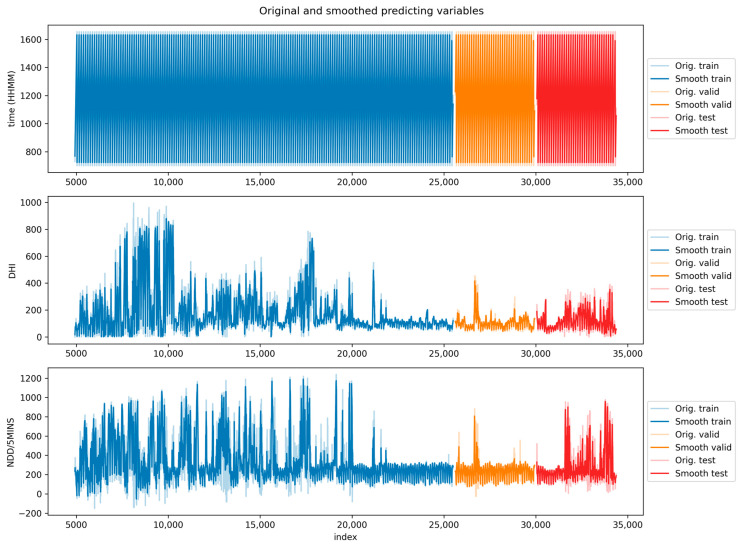
Location of the predictors’ training/validation/test sub-sets (blue/orange/red) corresponding to the target sub-sets shown in Figure 4. The top, middle, and bottom panels correspond to the time, DHI, and NDD/5 min predictors, respectively.

As an example, Figure 4 shows the training/validation/test sub-sets for the third folding of our five-fold ROCV, along with the whole solar dataset. The corresponding sub-sets for the predictors are displayed in Figure 5 where, along with each series, its smoothed version is also displayed, obtained as described in Section 4.1.

### 4.1. Data Preprocessing

Data preprocessing for both the wind and solar datasets was composed of the steps detailed in this section. However, these preprocessing steps were subject to the ROCV protocol, meaning that not all processes were necessarily applied and that their hyperparameters were selected as part of the model selection, along with the regressors’ selection. In other words, the model selection procedure selected both the best regressor and the best preprocessing steps (along with the relevant hyperparameters). What follows is therefore the list of potential preprocessing that the best model may have included, with the sole exception of windowing and normalization (which were applied in all cases).

#### 4.1.1. Normalization

Data have been scaled using a standard scaler, i.e.,

(2) $$x^{\prime }=\;\frac{x\;-\mu_{x}}{\sigma_{x}}$$

where *μ_x_* is the sample mean, and *σ_x_* is the standard deviation of the sample (both the predictors and the target variables were normalized).

#### 4.1.2. Windowing

The train/validation/test series were individually split into “predictor” and “target” windows. For the wind forecast task, the problem was framed as an autoregression task; hence, the predictor and the target variable effectively coincided. Notice, though, that the predictor window always preceded the corresponding target window. More specifically, the predictor and target windows covered a whole day and the subsequent one, respectively. As described above, for the wind dataset, the data sampling rate was 1 h, which automatically resulted in windows composed of 24 data points. Similarly, the solar dataset had sampling every 5 min but only during the irradiated hours, which ultimately resulted in windows composed of 120 data points. The stride between consecutive windows was 1 data point.

Windowing the dataset as just described resulted, for each folding, in the data shape summarized in Table 5. All the following procedures have been applied to windowed data.

#### 4.1.3. Smoothing

Smoothing consisted of replacing the data values with the centered rolling average of “win_smooth” data points, where “win_smooth” is a variable hyperparameter picked as part of the model selection protocol. The values of the points at the edge of a window (where fewer “win_smooth” data points were available) were left untouched. Smoothing was intended to reduce noise and enhance the signal of the underlying causal processes. Only the predictor variables were smoothed, as a smoothing of the target variable would have caused a decreased resolution in the forecast.

#### 4.1.4. Imputation

Missing values were imputed using scikit-learn’s SimpleImputer, which is a univariate imputer adopted to replace Nan values with the minimum value encountered at that time stamp position in any window. However, these replacements were extremely rare as missing values were only due to slight irregularities in the data sampling (usually at the end or the beginning of a day), and hence, their impact on the model was negligible. In this sense, value imputation was effectively implemented just to avoid running into numerical exceptions during execution.

### 4.2. Regressors Pool

The regressor constitutes the core of the pipeline; it was the estimator that learned to predict the preprocessed target variable from the preprocessed predictor variables. For this study, a large collection of regression algorithms was considered, including both “classical” regressors and deep neural network ones, namely,

- Classical regressors: linear regression [53], LightGBM (LGB [54]), and multi-layer perceptron (MLP [55]);
- Deep neural network regressors: multi-channel CNN (MCCNN [56,57]), distributed LSTM (DLSTM [58]), multi-head CNN (MHCNN [56,57]), LSTM [56,57], and WaveNet [59].

In the remainder of this section, the relevant information regarding the specific properties of the regressors and the choice of hyperparameters and architecture is provided. Consider that all algorithms were utilized in their multi-output form (except for LGB; see description) since the target variable was the array of values within the forecasted window. Notice that the architectures for the deep NNs were selected by trial and error and were not subject to auto-tuning in the CV loop.

#### 4.2.1. Linear Regression

This was a simple linear regression, without any regularization, with a bias term. This algorithm was introduced in the pool as a baseline comparison.

#### 4.2.2. LightGBM (LGB)

LGB is a tree-based gradient-boosting algorithm. The hyperparameters decided to tune in the CV loop were the number of tree estimators, the maximum number of leaves in a tree, and the learning rate. This was the only regressor that was not natively constructed to predict multi-output. Hence, in order to adapt it to our task, a custom multi-output estimator was created using scikit-learn’s MultiOutputRegressor wrapper. Notice that this meant learning an individual LGB regressor for each time step in the target window (e.g., if the task was to predict 24 time steps, the wrapper trained 24 LGB regressors).

#### 4.2.3. Multi-Layer Perceptron (MLP)

The MLP is a “vanilla” feedforward neural network, composed by dense layers with non-linear activation functions. Shallow architectures were examined, with either three layers composed of 500 neurons each or four layers composed of 200 neurons.

#### 4.2.4. Multi-Channel CNN (MCCNN)

Multi-channel CNNs, in this context, were neural networks that adopted one-dimensional convolutional layers, followed by flattening and dense layers. Here, the “multi-channel” attribute referred to the fact that they could synchronously parse multiple, separate input channels (Table 6). For the case of the solar dataset, the channels were time, DHI, and NDD(h,d), while for the wind case, being an autoregressive task, this network functioned as single-channel. 

#### 4.2.5. Distributed LSTM (DLSTM)

DLSTM refers to NNs composed of multiple LSTM cells followed by dense layers. Here, the “distributed” attribute referred to the fact that the LSTM cell returned the complete sequence of outputs (and not only the last) for each input sequence. This adjective was added to stress the difference from the LSTM framework described below. The chosen architecture is reported in Table 7.

#### 4.2.6. Multi-Head CNN (MHCNN)

**Figure 6:**
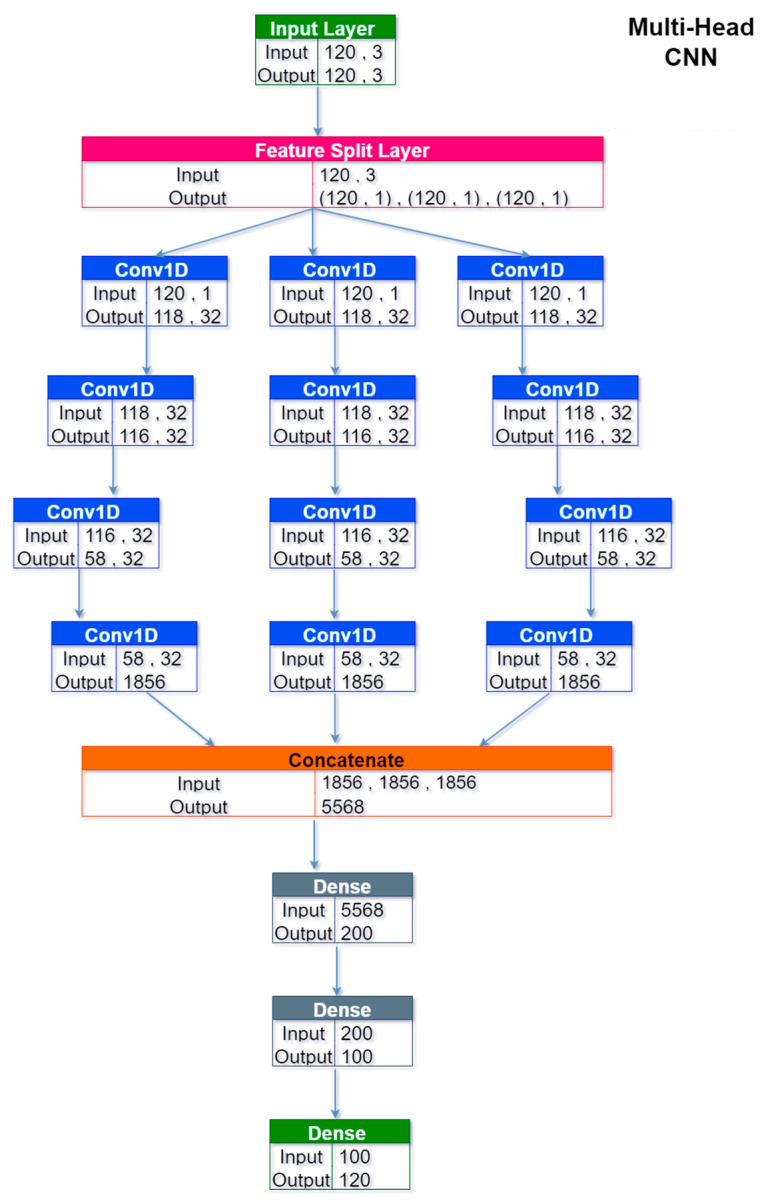
Architecture of the multi-head CNN for the solar forecasting task. For the wind task, the architecture reduced to a sequential CNN (with 24 input/output nodes instead of 120).

Multi-Head CNNs split the input features along parallel convolutional blocks, which learned separate weights for each channel of the data, as shown in the top part of Figure 6. The different heads were then concatenated before the dense layers. This architecture was effectively meaningful only for the solar forecasting task; as for the wind autoregressive task, it simplified back to the single-channel MCCNN (although with a different layer outlay).

#### 4.2.7. LSTM

This network was composed of sequential LSTM cells, followed by dense layers. This NN differed from the DLSTM presented above in the aspect that the last LSTM cells were not time-distributed, but they returned only the last output. The chosen architecture is reported in Table 8.

#### 4.2.8. WaveNet

**Figure 7:**
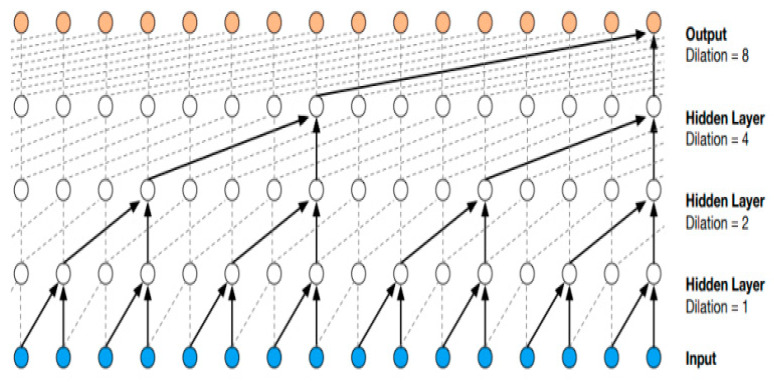
The dilated convolution rationale on which WaveNet is based shows the stack of causal convolutional operations.

**Figure 8:**
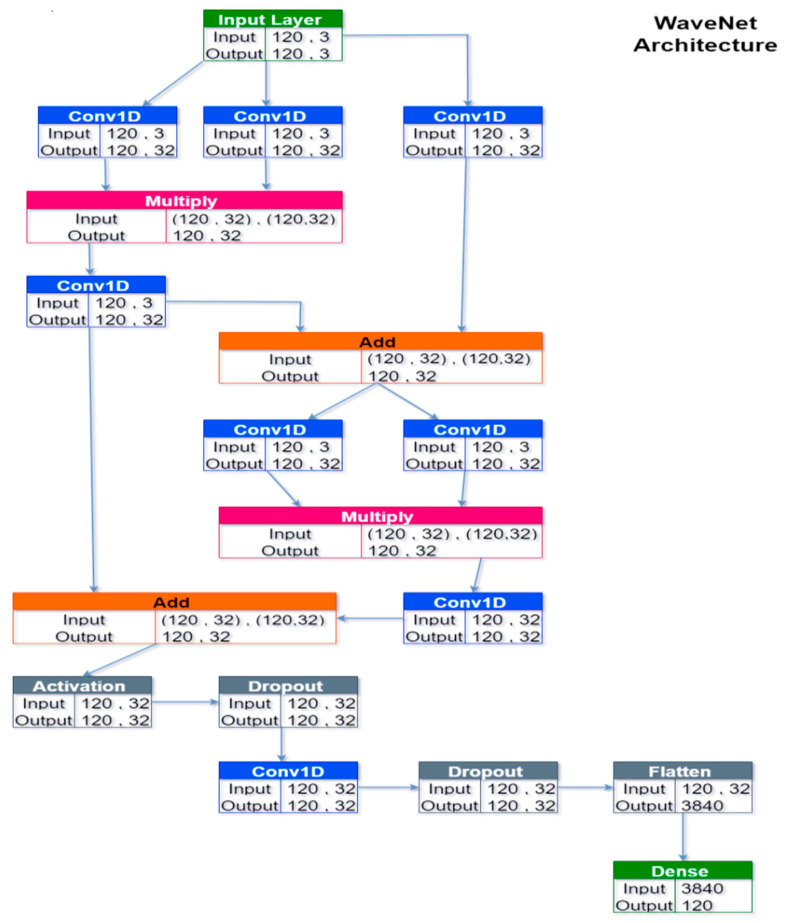
Architecture of the WaveNet for the solar forecasting task.

WaveNet was originally developed to analyze audio data (hence the name), but it was later successfully applied to generic forecasting tasks, notably wind speed forecasting [60]. This NN’s core is based on the concept of stacked, dilated causal convolutional layers (Figure 7). In brief, the main property was to present temporally wide receptive fields without sacrificing input resolution or computational speed, thanks to the relatively fewer layers/parameters necessary to the architecture (with respect to a convolutional network that uses standard convolution operations). In practice, WaveNet’s architecture was a collection of convolutional residual blocks and skipped connections; the specific architecture designed is graphically represented in Figure 8 for the solar forecasting task. For the wind task, the architecture was the same except for using 24 input/output nodes instead of 120 and one input feature instead of three.

For this regressor, the number of residual blocks (one or three) and the number of filters in the convolutional layers (16 or 32) were cross-validated.

### 4.3. Training Setup

To assess the model performance, the mean absolute error (MAE) and the root mean squared error (RMSE) were monitored; however, only the RMSE was used in order to perform the model selection. The RMSE was preferred as it was more sensitive to outliers, which—as evident from the top panels of Figure 1 and Figure 2—were particularly abundant in our datasets. Additionally, RMSE incorporated both bias and dispersion (variance), and hence, it was most suited for a model comparison task such as the one outlined in this section.

The NNs were trained using an Adam optimizer, whose learning rate was subject to CV along with the other model parameters. Given the abundance of data, a batch size of 32 samples was chosen for all the cases; this size provided a good balance between computational speed and accuracy in the estimation of the gradient. The number of training epochs was not selected a priori, as we preferred to adopt early stopping with a conservative threshold; in this regard, it was observed that most NN fits stopped within 10–20 epochs.

## 5. Results

### 5.1. Selected Models and Predictions

**Figure 9:**
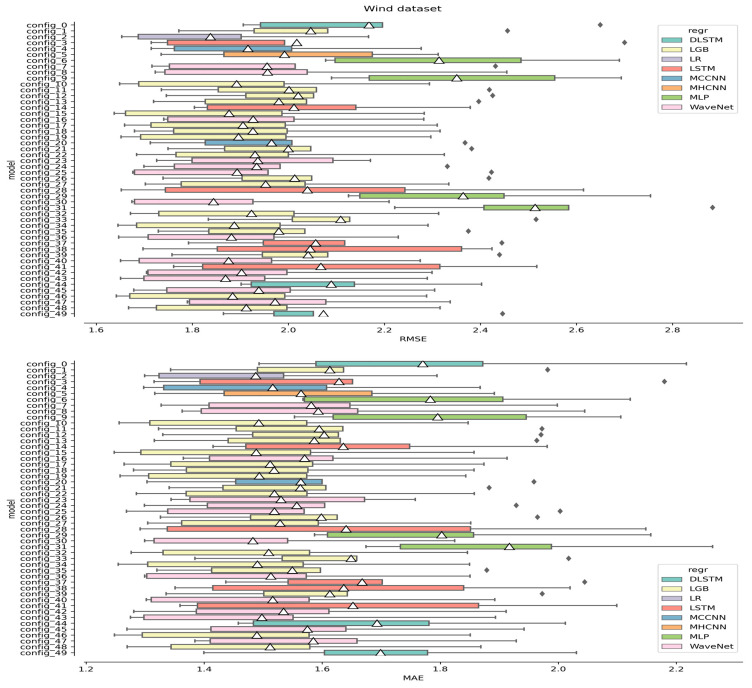
Results of the ROCV procedure for the wind dataset. The top panel presents the results assessed using the RMSE metric, while the bottom panel refers to the MAE metric.

**Figure 10:**
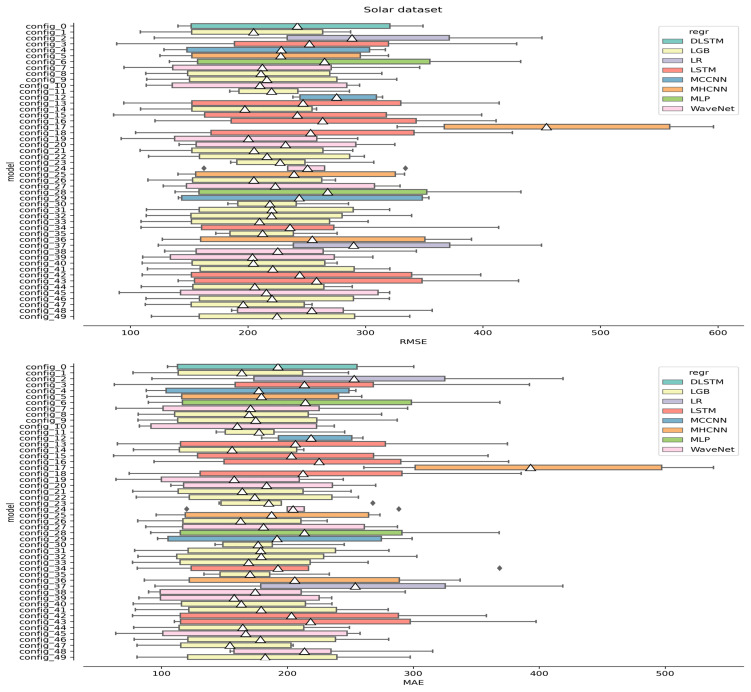
Results of the ROCV procedure for the solar dataset. The top panel presents the results assessed using the RMSE metric, while the bottom panel refers to the MAE metric.

In our ROCV procedure, 50 different model configurations were explored, selected at random by sampling within the hyperparameter ranges. Concerning the “model configuration”, it referred to all the essential hyperparameters that outlined the entire pipeline, encompassing both any preprocessing steps and the regressor. The scores measured for each configuration across the five different CV foldings are displayed in Figure 9 and Figure 10 for the wind and solar datasets, respectively. In either panel of Figure 9 and Figure 10, each box shows the interquartile range (IQR) of the distribution of scores registered for a given configuration, with the lines that extend from the IQR’s margins spreading outward by a factor of 1.5. The circles indicate the outliers, and the white triangle represents the mean value. Model configurations associated with the same regressor are color-coded with the same color. Better-performing configurations lean towards the left side.

Notice that the LGB (in orange) and WaveNet (in gray) regressors are over-represented in Figure 9 and Figure 10. This is simply because they had more hyperparameters to tune, which resulted in more frequent random sampling from our hyperparameter grid.

**Figure 11:**
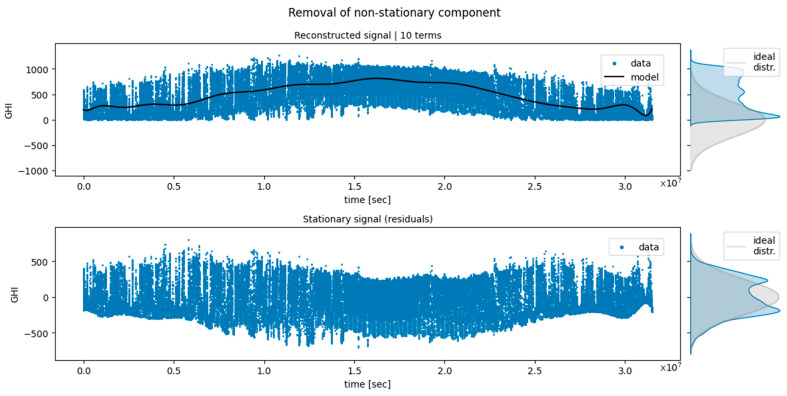
Results of the Lomb–Scargle model fitting (top) and subtraction (bottom) applied to the whole solar dataset.

Following the analysis of Figure 9 and Figure 10, it can be observed how the models showed, with few exceptions, comparably large variance. This was surprising considering that the regressors used were based on significantly different algorithms. We related this effect to the complexity of the data: no configuration could really adapt better than the others, and they all resulted in large variances. In particular, the wind data were extremely irregular, with no clear dominant periodicity (Figure 1). On the other hand, the solar dataset showed the obvious seasonal variation, but since the dataset only contained one single trend oscillation, this could not be properly modeled within one CV folding (each training set covered about half a period; see Figure 4). To try and bypass the latter issue, an attempt was made to fit and subtract a Lomb–Scargle (L-S) model before windowing the data with the aim of removing the non-stationary component. As a depiction of this procedure, Figure 11 shows the application of L-S to the whole solar dataset. Notice that this depiction represents an edge case since, during CV, L-S has access to even less training data. On the top of Figure 11, a 10-component L-S model (black line) was fitted to the data (blue). The curves on the right side of the panel show the distribution of the data (blue) and the ideal distribution one would like to obtain if any non-stationarity were to be removed. On the bottom of Figure 11, the model-subtracted data and relevant distributions are presented. However, as also evident from the residuals in Figure 11, a seasonal-free signal was not obtained due to the large daily fluctuations in the data, and hence, this procedure was discarded.

**Figure 12:**
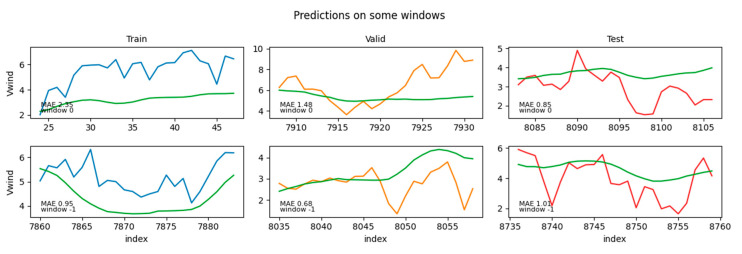
Example predictions for the wind dataset.

**Figure 13:**
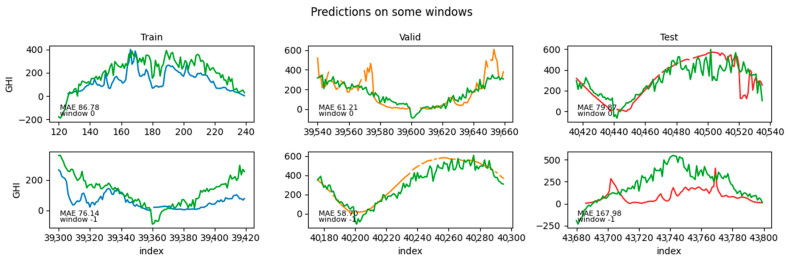
Example prediction dataset for the solar dataset.

The best-fit models selected by the procedure outlined in this section included, as regressors, LR for the wind speed forecasting and LGB for the solar irradiance forecasting. The performance metrics evaluated over the hold-out test set are reported in Table 9, while some example predictions are shown in Figure 12 and Figure 13 for wind speed and solar irradiance, respectively. In Figure 12 and Figure 13, each panel displays the data and the model prediction (in green) for a given target window in the train (blue; left), validation (orange; center), and test set (red; right). The top row shows the results for the first window in the train/validation/test sets, while the bottom row shows the results for the last window in the corresponding sets. In each panel, the MAE for that window is also reported, while the MAE averaged across all test windows is reported in Table 9.

Figure 12 and Figure 13 shows examples of predictions per window, as forecasted from the immediately preceding predictors’ window (which has a size <*n_features*>). Consider, though, that forecasting windows overlapped because each window was obtained by shifting the previous by one time step. That, in turn, implied that each target time step *t* possessed multiple predictions, one from each target window that encompassed it. Namely, each individual time step in the wind (/solar) dataset had 24 (/120) predictions.

Intuitively, for a given target time step *t*, the predictors’ window immediately adjacent—i.e., from time step (*t* − *n_features*) to (*t* − 1)—was expected to yield the best prediction simply because less extrapolation into the future was needed. In practice, though, this was not strictly true, as it will be shown in the Section 5.2 “*Feature importance analysis*”. It was therefore desired to inspect the confidence intervals of a prediction at a given time step *t*, assuming that we ignored whether the predictors’ window provided at inference time was immediately adjacent or anywhere in the possible range—i.e., between (*t* − 2 × *n_features*) and (*t* − 1).

**Figure 14:**
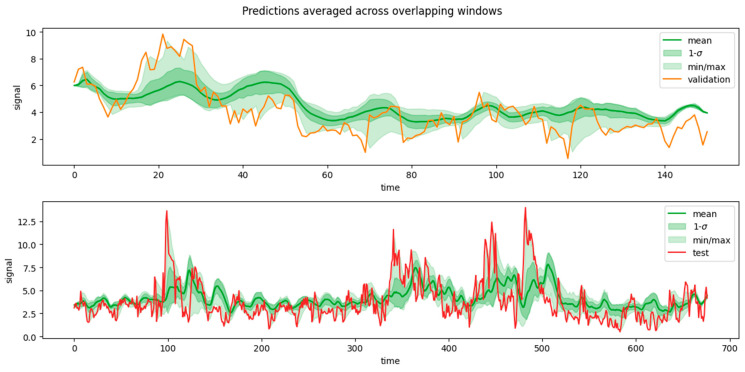
Model predictions along the whole test set (top) and validation (bottom) set for wind speed.

**Figure 15:**
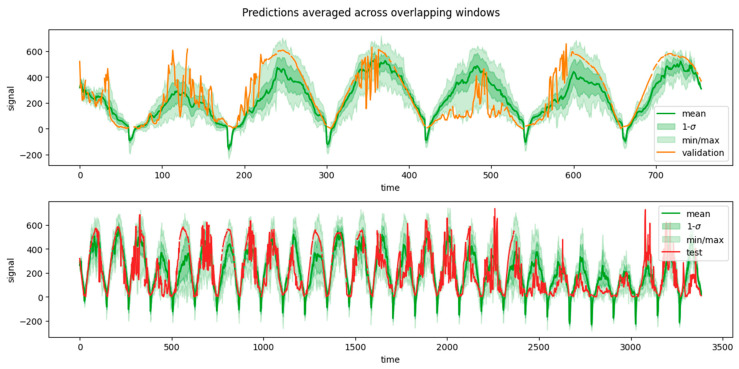
Model predictions along the whole test set (top) and validation (bottom) set for solar irradiance.

In order to obtain such a summary picture of the predictions along the whole set, the predictions of individual windows were combined into one single series, as shown in Figure 14 and Figure 15. This combination was obtained by recording the mean value of the predictions at each time step (coming from multiple prediction windows), along with their standard deviations, as well as their min/max predictions. In Figure 14 and Figure 15 the solid green curve represents the mean value of the prediction at each time step, while the dark shaded area represents its standard deviation. The min/max predictions are represented by the light shaded area. In general—but not always—the prediction window closest to a given target time step *t* was the one yielding the best prediction (minimal deviance from the true value within the shaded area), and vice versa for the window farthest in time (maximal deviance from the true value). 

### 5.2. Feature Importance Analysis

In the context of time series forecasting, “feature” refers to the individual time steps inside a predictor window, and “feature importance” refers to the impact that such a feature had in determining the predicted target value. The feature importance for the selected models was explored using the SHAP (Shapley Additive Explanations) library [61] (in particular, for LightGBM, its TreeExplainer [62] implementation). In brief, the SHAP value estimated the impact of a given feature f on the model’s prediction, and it was calculated by comparing the prediction value when f was present, against the values predicted when considering all possible combinations of features. In regression problems, a SHAP value effectively equals how much of a sample’s prediction diverges from the average prediction (across samples) due to its specific value of f, with negative values indicating a decrease with respect to the mean prediction and vice versa. In other words, the SHAP value denotes how much a specific feature pushes the predictor away from the average target value. A SHAP value can be averaged across samples to obtain a mean estimate of the contribution of a feature. In the present analysis, the absolute value of the mean SHAP score was considered, as we were interested in the global contribution of a feature, regardless of whether this pushed the prediction higher or lower. In the remainder, this quantity will be referred to interchangeably with the more generic label “SHAP Value”.

**Figure 16:**
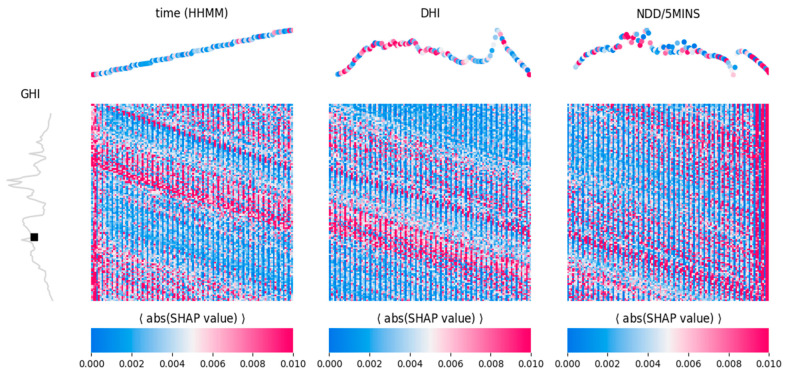
SHAP heat maps relative to solar task.

**Figure 17:**
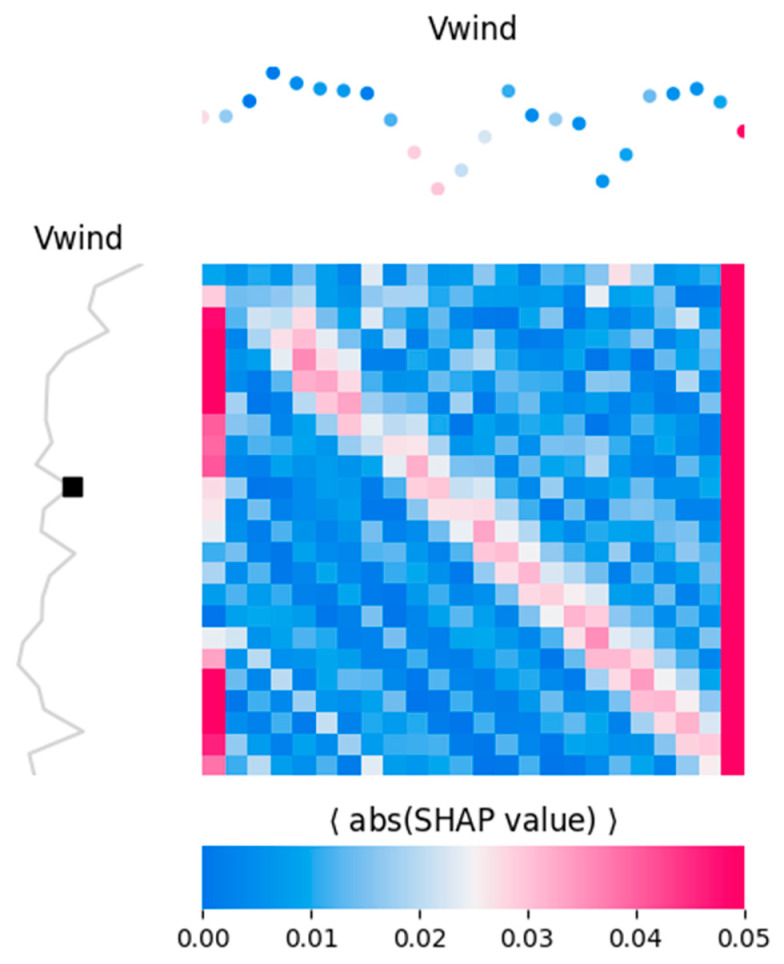
SHAP heat maps relative to wind task.

Given that a predictor sequence was composed of time steps (120 for the solar dataset and 24 for the wind dataset), a SHAP value was calculated for each single time step along that sequence. Consider that target windows composed of time steps were forecasted, and hence, (*n_features* × *n_features*) SHAP values were provided, i.e., one SHAP value per target time step, per predictor time step. This information could therefore be summarized in a heat map, as shown in Figure 16 and Figure 17. In Figure 16 each heat map refers to a predictor (time, DHI, and NDD), with the color intensity related to the mean absolute SHAP value. On the x-axis are the predictor features (one for each of the 120 input time steps), while on the y-axis are the targets (one for each of the 120 target time steps). A given row represents a single time step along the 120-target sequence, and the color intensity along that row (i.e., for that time step) shows the corresponding importance of the predictor features in influencing the prediction of that target value. The curve plots around the heat maps are purely illustrative; they represent a “typical” example of a predictor (top) or target (left) window and are used to indicate where a heat map column or a row lies along a predictor/target window. The heat maps are averaged across all the samples, while these curves represent a single sample (window), and a single time step is just a point along those curves. For example, the black squared dot indicates the 50th time step along the target window. Figure 17 is the heat map for wind speed forecasting (one predictor and 24 target time steps).

The heat maps of Figure 16 and Figure 17 shall be interpreted as follows: Consider the heat map for the solar task (Figure 16) and let us assume we want to inspect what influences, on average, the prediction of the 50th target time step. This can be implemented by focusing on the curve plots around the heat maps, which are intended exactly for the inspection of a specific target time step. The curve plot on the left panel shows a representative example of a “typical” target window, but it can be used to indicatively locate the 50th target time step along any window: the black squared dot. Let us now infer which DHI feature affects the most this 50th time step, on average, by looking at the plot over the corresponding heat map (central top panel). This is also just a representative example of a “typical” predictor window. It is color-coded by the importance of each predictor time step (i.e., the same color as the row corresponding to the black square in the heat map). It is observed that DHI time steps 0–60 tended to impose a larger impact on the prediction, while time steps 61–120 did the opposite (the more intense the color, the larger the SHAP value, and the stronger the influence of that predictor time step).

## 6. Discussion

It is worth noting that almost every model showed improved forecasting performance except for MLP in wind speed forecasting and MHCNN in solar irradiance forecasting (see Figure 9 and Figure 10). It was interesting to notice how LR performed so efficiently for wind speed forecasting, despite being such a simple mode, although the difference from LGB and WaveNet was marginal (see Figure 9).

Moreover, according to the heat map in Figure 16, it is obvious that time step 0 in time (HHMM) and time step 120 in NDD/5MINS have always had a huge impact on solar irradiance forecasting. The slanting red patterns in Figure 16 are for time (HHMM).

DHI and NDD/5 min showed that every forecasting time step was correlated with the corresponding time step in the predictor window. For instance, forecasting time step number 50 was correlated with the 50th time step on the predictors window. This happened due to the relative periodicity of solar irradiance.

For wind speed forecasting, the heat map in Figure 17 shows that the 50th time step on the predictor window had a huge impact, which was arguably due to the large variability of wind. In Figure 17 the faint slanting red patterns were indicative of the relative correlation between forecasting time steps and the corresponding time steps of the predictors window. This was due to the relatively small periodicity of wind speed during a year.

## 7. Summary and Conclusions

In this paper, a variety of different model configurations were constructed and employed to generate wind speed and solar irradiance forecasts for measurements conducted at the Laboratory of Energy and Photovoltaic Systems (LEPS) of Hellenic Mediterranean University (HMU) in Crete, Greece. Almost all the models showed improved forecasting performance. For wind speed forecasting, LR showed the best performance, whereas in solar irradiance forecasting, LGB showed the best performance. Moreover, an integrated methodology was followed in this research, containing data preprocessing, CV and model selection, average prediction presentation, and an extensive feature importance analysis.

It is important to mention that regarding the feature importance analysis, wind speed was best predicted by the wind speed at the first and last hour of the predictor window (the previous day) and secondarily by the wind speed at the same hour of the predictor window (the previous day). For the solar irradiance, the situation was more complex and was affected mainly by a different range of hours from the predictor window (the previous day).

Accurate medium-term forecasts of solar irradiance and wind speed enable wind farms and solar plants to anticipate their power output for the next day, improving the scheduling of generation and guaranteeing the effective integration of generated electricity into the grid. Moreover, medium-term forecasts can handle the ideal period to charge or discharge batteries, inform demand response strategies, and allow for the scheduling of maintenance during periods of low expected generation, minimizing the impact on the grid. Furthermore, accurate medium-term forecasts enable renewable energy producers to bid more effectively in energy markets, leading to better price discovery and increased revenue [63].

Developments in medium-term solar irradiance and wind speed predictions will be crucial to the development of power systems. Increasing accuracy and precision in one-day solar irradiance and wind speed forecasts provides grid operators with the ability to anticipate and balance energy output and consumption appropriately, particularly in isolated systems.

## Figures and Tables

**Table 1:** Dataset parameters measured.

Parameter	Unit
Day	
Time	
Air temperature	°C
Wind speed	m/s
Global irradiance on the horizontal plane	W/m^2^
Diffuse irradiance on the horizontal plane	W/m^2^
Extraterrestrial irradiance on the horizontal plane	W/m^2^

**Table 2:** Dataset max, min, mean, and Std values.

	Max	Min	Mean	Std
Global irradiance on the horizontal plane (W/m^2^)	1264.50	0	211.75	315.38
Wind speed (m/s)	18.40	0	4.51	2.58
Air temperature (°C)	37.70	1.90	17.64	6.38
Diffuse irradiance on the horizontal plane (W/m^2^)	995.30	0	61.85	105.06
Extraterrestrial irradiance (W/m^2^)	1294	0	344	429

**Table 3:** Wind forecasting categorization.

Very short term	Ranging from a few seconds to 30 min
Short term	Spanning from 30 min to 6 h ahead
Medium term	Extending from 6 h to 1 day ahead
Long term	Beyond 1 day ahead

**Table 4:** Solar irradiance forecasting categorization.

Very short term	Covering a time frame from a few minutes to 1 h
Short term	Encompassing a period of 1 to 4 h ahead
Medium term	Forecasting for 1 day ahead
Long term	Extending beyond 1 day ahead

**Table 5:** Shapes of windowed datasets within one folding.

Set	Wind Forecasting		Solar Forecasting	
	Predictors	Target	Predictors	Target
Train	(4092, 24, 1)	(4092, 24)	(20,456, 120, 3)	(20,456, 120)
Validation	(839, 24, 1)	(839, 24)	(4195, 120, 3)	(4195, 120)
Test	(841, 24, 1)	(841, 24)	(4197, 120, 3)	(4197, 120)

Note: Shapes are expressed as (n_samples, *n_features*, n_channels).

**Table 6:** MCCN architecture.

Wind Forecasting		Solar Forecasting	
Layer	Description	Layer	Description
Conv1D	3× (32), ReLu	Conv1D	3× (32), ReLu
Conv1D	3× (32), ReLu	Conv1D	3× (32), ReLu
MaxPool1D	2	MaxPool1D	2
Conv1D	3× (16), ReLu	Conv1D	3× (16), ReLu
MaxPool1D	2	MaxPool1D	2
Flatten	-	Flatten	-
Dense	24, Relu	Dense	120, Relu
Dense	24	Dense	120

**Table 7:** DLSTM architecture.

Wind Forecasting		Solar Forecasting	
Layer	Description	Layer	Description
LSTM	16, distr., tanh	LSTM	32, distr., tanh
LSTM	16, distr., tanh	BatchNorm	-
Dense	24, ReLu	LSTM	32, distr., tanh
BatchNorm	-	BatchNorm	-
Dense	12, ReLu	TimeDistrDense	32, ReLu
BatchNorm	-	BatchNorm	-
Dense	24	TimeDistrDense	1

**Table 8:** LSTM architecture.

Wind Forecasting		Solar Forecasting	
Layer	Description	Layer	Description
LSTM	16, distr., tanh	LSTM	32, distr., tanh
LSTM	16, tanh	LSTM	32, tanh
Dense	24, ReLu	Dense	64, ReLu
BatchNorm	-	BatchNorm	-
Dense	12, ReLu	Dense	64, ReLu
BatchNorm	-	BatchNorm	-
Dense	24	Dense	120

**Table 9:** Performance of the best-fit models.

	Wind Forecasting	Solar Forecasting
Regressor	LR	LGB
MAE	1.58	99.32
RMSE	2.18	135.68

## Data Availability

The dataset presented in this article is not readily available because the data are part of an ongoing study.
